# Supplementary material for: Dosage sensitivity is a major determinant of human copy number variant pathogenicity
Source: Nat Commun. 2017 Feb 8;8:14366. doi: 10.1038/ncomms14366 (PMC5309798; doi:10.1038/ncomms14366)
Supplement: Supplementary Information — Supplementary Figures, Supplementary Table, and Supplementary References [file ncomms14366-s1.pdf]

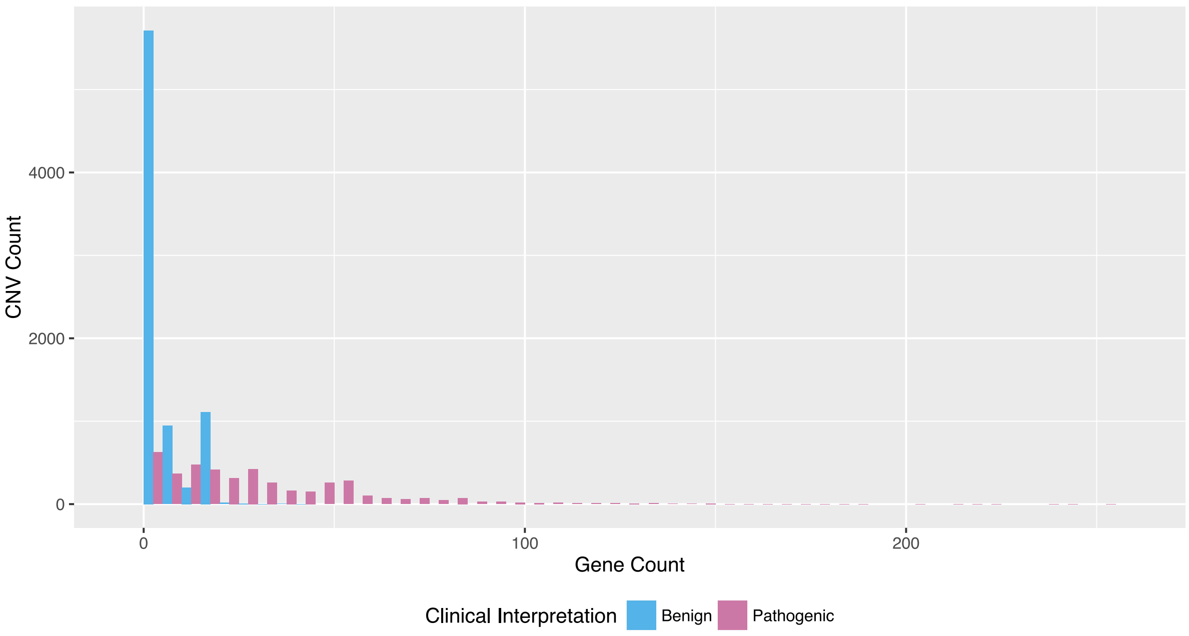

Figure 1: Number of genes per copy number variant (CNV) in dataset
Histogram of the number of genes per CNV separated by CNV clinical interpretation (benign/pathogenic). Bin width set to 5.

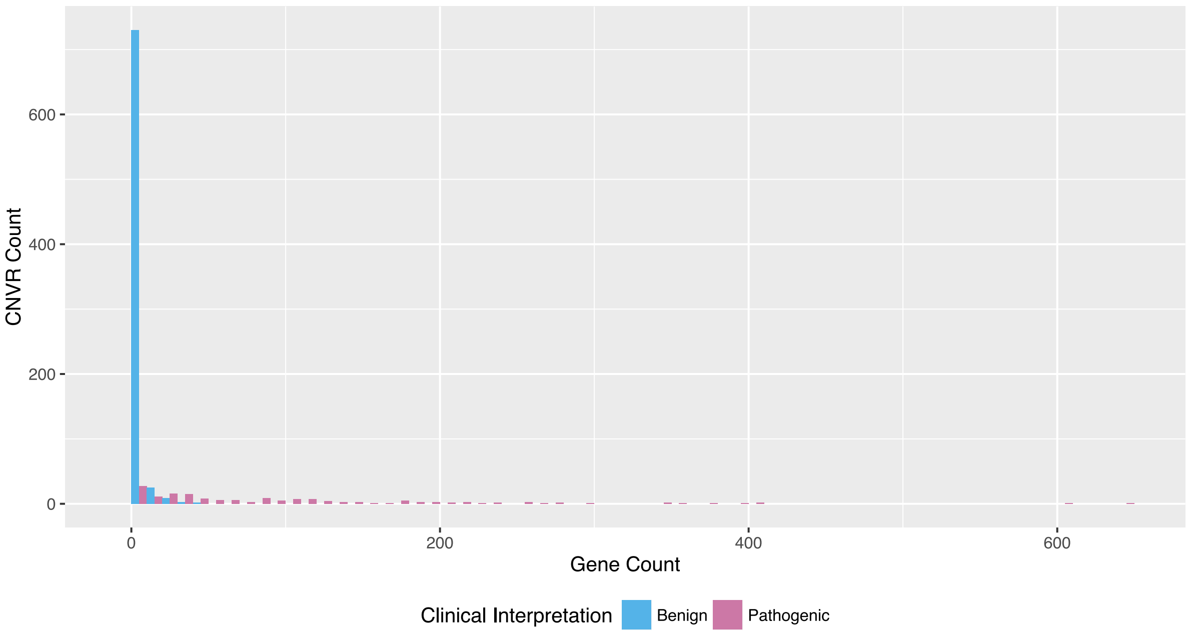

Figure 2: Number of genes per CNV region (CNVR)
Histogram of the number of genes per CNVR separated by CNV clinical interpretation (benign/pathogenic). Bin width set to 10.

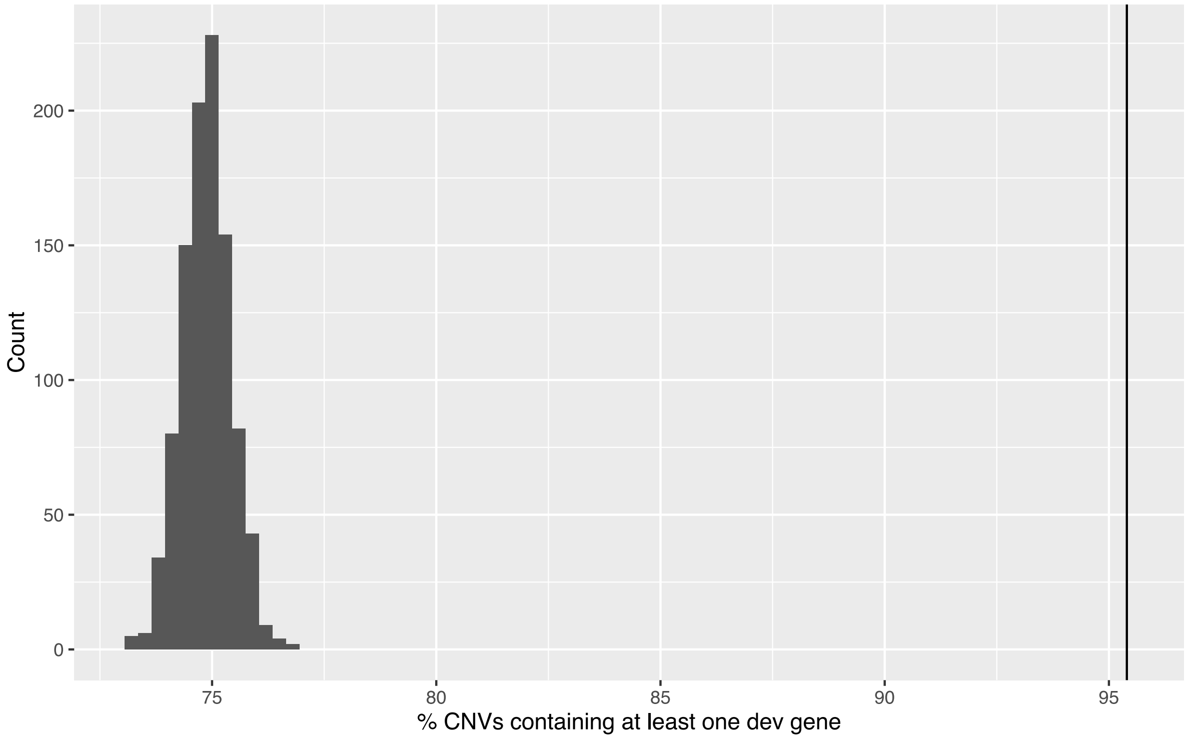

Figure 3: Percentage of CNVs containing at least one developmental gene for 1000 randomised sets
Pathogenic CNV positions were shuffled randomly 1000 times, after each the percentage of CNVs that contain at least one developmental gene was calculated. The observed value for dbVar pathogenic CNVs is overlaid as a black line. Bin width set to 0.3.

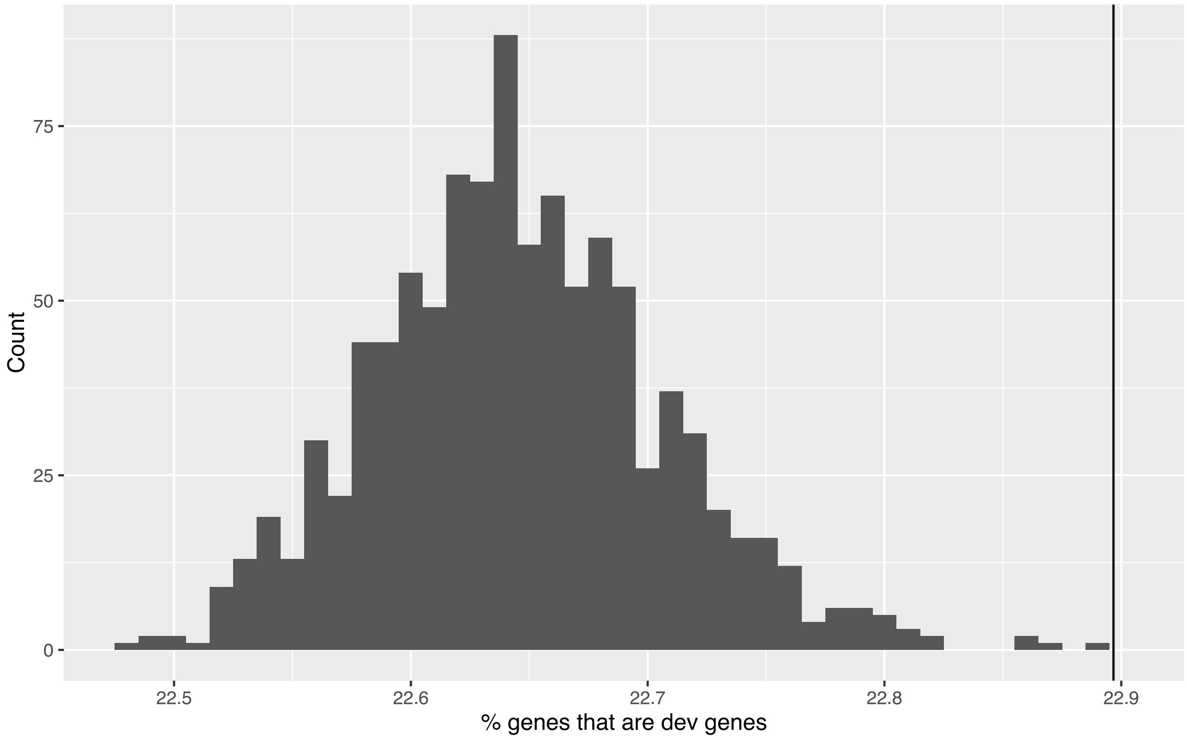

Figure 4: Percentage of developmental genes overlapped by each randomised CNV set
Pathogenic CNV positions were shuffled randomly 1000 times, after each the percentage of developmental genes overlapped was calculated. The observed value for dbVar pathogenic CNVs is overlaid as a black line. Bin width set to 0.01.

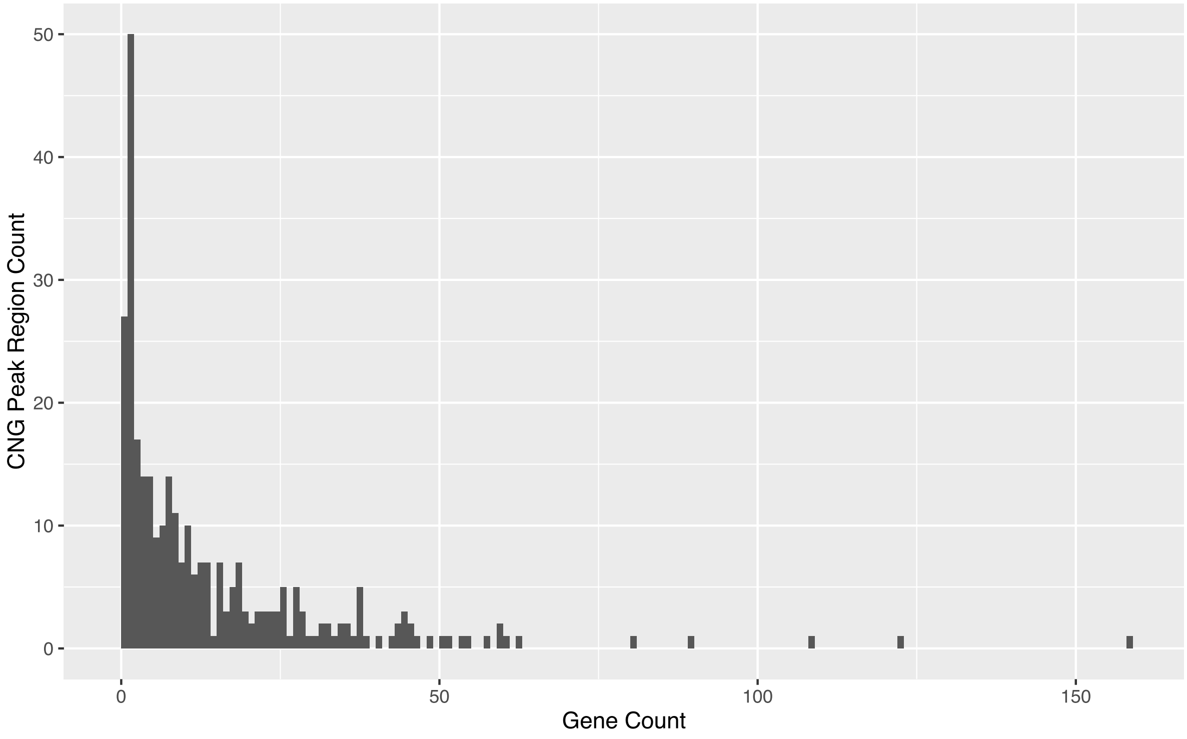

Figure 5: Number of Class P genes per copy number gain (CNG) peak region
Histogram of the number of Class P genes per CNG peak region. Bin width set to 1.

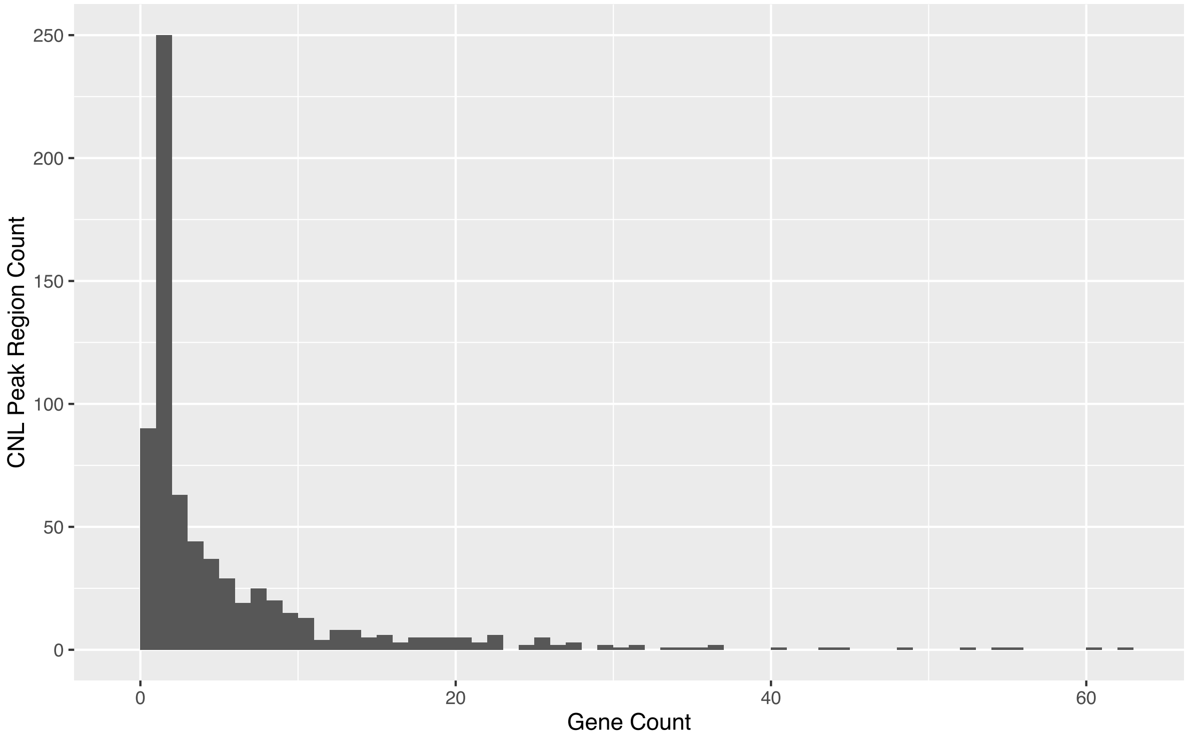

Figure 6: Number of Class P genes per copy number loss (CNL) peak region
Histogram of the number of Class P genes per CNL peak region. Bin width set to 1.

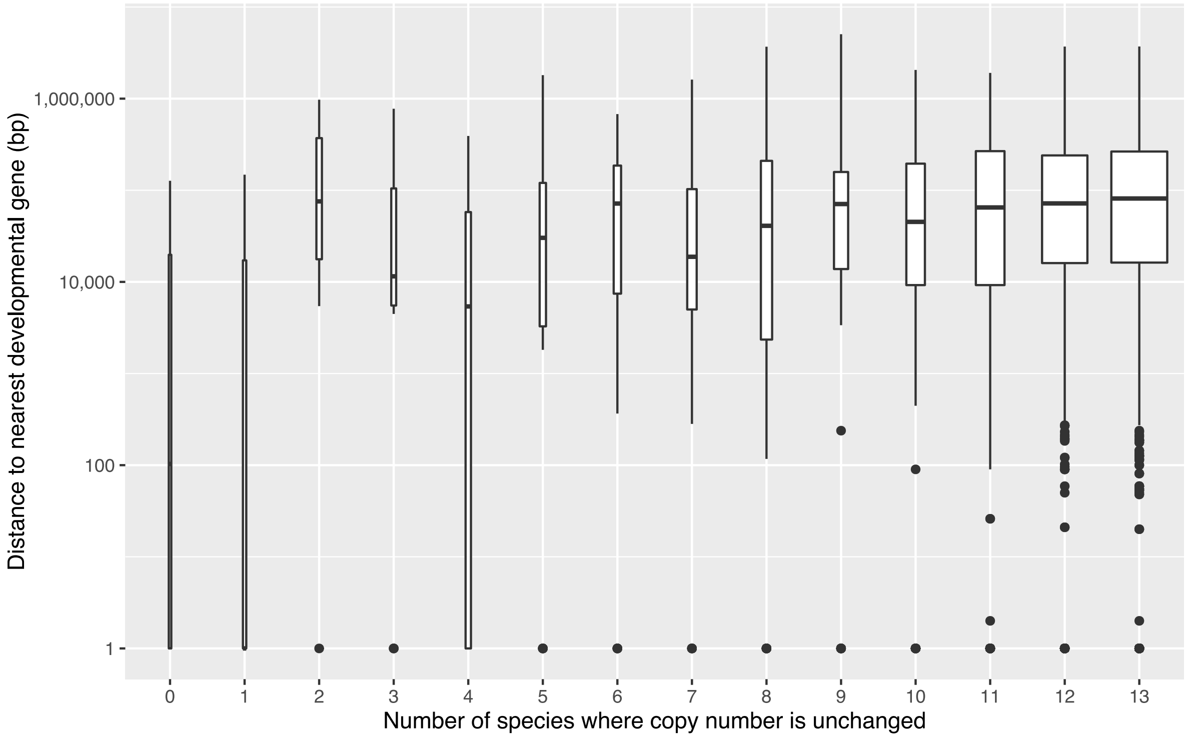

Figure 7: Distance to nearest developmental gene for developmental genes grouped by number of genomes where ortholog has unchanged copy number
For each developmental gene, distance to the closest upstream or downstream developmental gene in base pairs was calculated, ignoring strand. For developmental genes that overlap a distance of 1 base pair was assigned. Developmental genes are grouped by the number of genomes where ortholog copy number is unchanged (13 where a gene has a one-to-one relationship with all 13 mammalian genomes tested). Width of each box is proportional to sample size in each group and the median is shown within each box. Upper and lower hinges of boxes correspond to the first and third quartiles. Whiskers extend to values 1.5 * interquartile range.

Table 1: dbVar studies included in CNV analysis

Study	Number of CNVs included	Reference
Miller et al. [1]	7,586	[1]
Kaminsky et al. [2]	2,507	[2]
Wapner et al. [3]	1,773	[3]
Mitsui et al. [4]	173	[4]
Riggs et al. [5]	67	[5]
dbVar user submitted curated variants from OMIM, GeneReviews, or ClinVar	34	[6]
Sharp et al. [7]	9	[7]
Zhang et al. [8]	6	[8]
Sharp et al. [9]	4	[9]
Lopez-Herrera et al. [10]	1	[10]
CNVs lacking study IDs in 2016	211	[6]

References

[1] David T Miller et al. "Consensus Statement: Chromosomal Microarray Is a First-Tier Clinical Diagnostic Test for Individuals with Developmental Disabilities or Congenital Anomalies". In: *The American Journal of Human Genetics* 86.5 (Feb. 2011), pp. 749–764.

[2] Erin B Kaminsky et al. "An evidence-based approach to establish the functional and clinical significance of copy number variants in intellectual and developmental disabilities." In: *Genetics in medicine : official journal of the American College of Medical Genetics* 13.9 (Sept. 2011), pp. 777–784.

[3] Ronald J Wapner et al. "Chromosomal microarray versus karyotyping for prenatal diagnosis." In: *The New England journal of medicine* 367.23 (Dec. 2012), pp. 2175–2184.

[4] Jun Mitsui et al. "Mechanisms of genomic instabilities underlying two common fragile-site-associated loci, PARK2 and DMD, in germ cell and cancer cell lines." In: *American journal of human genetics* 87.1 (July 2010), pp. 75–89.

[5] E R Riggs et al. "Towards an evidence-based process for the clinical interpretation of copy number variation." In: *Clinical genetics* 81.5 (May 2012), pp. 403–412.

[6] Ilkka Lappalainen et al. "DbVar and DGVa: public archives for genomic structural variation." In: *Nucleic acids research* 41.Database issue (Jan. 2013), pp. D936–41.

[7] Andrew J Sharp et al. "A recurrent 15q13.3 microdeletion syndrome associated with mental retardation and seizures." In: *Nature Genetics* 40.3 (Mar. 2008), pp. 322–328.

[8] Qian Zhang et al. "Combined immunodeficiency associated with DOCK8 mutations." In: *The New England journal of medicine* 361.21 (Nov. 2009), pp. 2046–2055.

[9] Andrew J Sharp et al. "Characterization of a recurrent 15q24 microdeletion syndrome." In: *Human molecular genetics* 16.5 (Mar. 2007), pp. 567–572.

[10] Gabriela Lopez-Herrera et al. "Deleterious mutations in LRBA are associated with a syndrome of immune deficiency and autoimmunity." In: *American journal of human genetics* 90.6 (June 2012), pp. 986–1001.
